# HHV-6A infection induces amyloid-beta expression and activation of microglial cells

**DOI:** 10.1186/s13195-019-0552-6

**Published:** 2019-12-12

**Authors:** Daria Bortolotti, Valentina Gentili, Antonella Rotola, Elisabetta Caselli, Roberta Rizzo

**Affiliations:** Department of Chemical and Pharmaceutical Sciences, University of Ferrara, Via Luigi Borsari, 46, 44121 Ferrara, Italy

**Keywords:** Alzheimer’s disease, Microglia, Infection, Apolipoprotein-E, Beta-amyloid

## Abstract

**Background:**

The control of viral infections in the brain involves the activation of microglial cells, the macrophages of the brain that are constantly surveying the central nervous system, and the production of amyloid-beta (Aβ) as an anti-microbial molecule. Recent findings suggest a possible implication of HHV-6A in AD. We evaluated the effect of HHV-6A infection on microglial cell expression Aβ and the activation status, determined by TREM2, ApoE, cytokines, and tau expression.

**Methods:**

We have infected microglial cells (HMC3, ATCC®CRL-3304), in monolayer and human peripheral blood monocyte-derived microglia (PBM-microglia) spheroid 3D model, with HHV-6A (strain U1102) cell-free virus inocula with 100 genome equivalents per 1 cell. We collected the cells 1, 3, 7, and 14 days post-infection (d.p.i.) and analyzed them for viral DNA and RNA, ApoE, Aβ (1-40, 1-42), tau, and phospho-tau (Threonine 181) by real-time immunofluorescence and cytokines by immunoenzymatic assay.

**Results:**

We observed a productive infection by HHV-6A. The expression of Aβ 1-42 increased from 3 d.p.i., while no significant induction was observed for Aβ 1-40. The HHV-6A infection induced the activation (TREM2, IL-1beta, ApoE) and migration of microglial cells. The secretion of tau started from 7 d.p.i., with an increasing percentage of the phosphorylated form.

**Conclusions:**

In conclusion, microglial cells are permissive to HHV-6A infection that induces the expression of Aβ and an activation status. Meanwhile, we hypothesize a paracrine effect of HHV-6A infection that activates and induces microglia migration to the site of infection.

## Introduction

Alzheimer’s disease (AD) is multifactorial and characterized by early neuronal loss. In AD brains, two pathological characteristics are observed: extracellular insoluble senile plaques formed by amyloid-β (Aβ) peptide and intraneuronal neurofibrillary tangles (NFT) formed by tau protein [1]. Aβ, thought to be primarily produced by neurons, can activate an inflammatory response that ultimately drives microglia and astrocytes to uptake and clear it from the brain [2, 3].

The elevation of intracellular soluble Aβ leads to an abnormal phosphorylation of tau that is relocated from axons to the somatodendritic compartments of neurons [4]. Here, tau can bind and sequester the Src tyrosine kinase, fyn, altering its localization [5] and the phosphorylation and stabilization of excitatory GluN2B NMDA receptors. This enhances glutamate signaling and causes an intracellular flood of Ca2+, which enhances Aβ toxicity [5–7]. Calcium-induced excitotoxicity can damage post-synaptic sites and cause mitochondrial Ca2+ overload, membrane depolarization, oxidative stress, and apoptotic cell death [6, 8].

Interestingly, Aβ plaques and NFT are not unique to AD. Other central nervous system conditions, including chronic infections, develop with the production of these specific histopathologic hallmarks [9]. Recent studies show the possibility that infections may be associated with AD and indicate the crucial role of neuroinflammation in the etiopathogenesis of AD [10, 11]. The inflammatory response is a typical reaction during most infectious diseases that might stimulate activation of microglia, specialized macrophages resident in the central nervous system (CNS), where they remove damaged neurons and infections. Recent findings have shown that infections may induce Aβ production and deposition in the brain with an anti-microbial activity [11]. Aβ fibrilization pathway seems to be an innate immune response that protects against viral, fungal, and bacterial infections. Aβ oligomers bind herpesvirus surface glycoproteins, accelerating β-amyloid deposition and leading to protective viral entrapment activity in 5XFAD mouse and 3D human neural cell culture infection models against neurotropic herpes simplex virus 1 (HSV1) and human herpesvirus 6A and B [12]. HHV-6 has been examined for its potential role in AD pathogenesis [10, 13]. Human herpesvirus 6 (HHV-6) is a betaherpesvirus that exists as two closely related species, HHV-6A and HHV-6B [14]. HHV-6A has not been etiologically linked to any disease; HHV-6B is the causative agent of exanthema subitum (ES), a childhood disease characterized by high fever and a mild skin rash, occasionally complicated by seizures or encephalitis. The term HHV-6 remains in usage and collectively refers to the two species. HHV-6 exhibits wide cell tropism in vivo and, as with other herpesviruses, induces a lifelong latent infection in humans. HHV-6 preferentially replicates in activated CD4+ T lymphocytes [15, 16] and uses specific cell receptors permitting virus anchorage to the cell surface: HHV-6A uses CD46, a regulator of complement activation expressed on all nucleated cells, while CD134 (also called OX40), a member of the tumor necrosis factor (TNF) receptor superfamily present only on activated T lymphocytes, functions as a specific entry receptor for HHV-6B [17, 18]. In vitro experimentation has shown that in addition to CD4+ T lymphocytes, HHV-6 can infect CD8+ T lymphocytes (only with HHV-6A), fibroblasts, natural killer (NK) cells [19], liver cells, epithelial cells, endothelial cells, microglial cells, astrocytes, and oligodendrocytes [15, 19–26]. The in vivo host tissue range appears to be broader than expected from in vitro studies and includes the brain, salivary glands, tonsils, kidneys, lymph nodes, liver, heart, gastrointestinal tract, lungs, and monocytes/macrophages [15, 27–29]. The preferential sites for latency are suspected to be monocytes/macrophages, bone marrow progenitors, and central nervous system cells [30–32]. Fecal-oral spread, a common transmission route among young children, has not been documented for HHV-6, although stool specimens were found positive for HHV-6 DNA [33]. Thus, the most probable route for HHV-6B transmission is through saliva [34]. Very little is known about the epidemiology of HHV-6A, which is acquired later in life, is not typically found in the saliva, and has an unknown mechanism of transmission. Interestingly, HHV-6 DNA can be integrated into the subtelomeric region of host chromosomes as an inherited, chromosomally integrated HHV-6 variant (iciHHV-6), which is present in about 1% of the general population and is passed through generations via vertical transmission [35].

Carbone and colleagues analyzed DNA isolated from PBL and brain samples for the presence of EBV, HHV-6, and CMV [36]. DNA of HSV1, EBV, and HHV-6, but not CMV, was found. Interestingly, HHV-6 was found in 70% of AD brains vs 40% of controls, while HSV-1 was found at high levels in both. In this prospective study, increases in EBV-positive or HHV-6-positive PBL were noted in patients who developed clinical AD. Readhead and co-authors have determined that genes involved with AD overlap with those involved in fighting viral infection [10]. They found HHV-6A and HHV-7 to be more abundant in Alzheimer’s brains and singled out HHV-6A as a key modulator of the genes involved in amyloidosis and neuronal death. HHV-6A and HHV-7 DNA transcription was increased among AD patients, and this was observed across multiple brain regions and multiple cohorts with a lower abundance in healthy aging controls. Host genes affected by HHV-6A were associated with Alzheimer’s traits and risk genes. Also, HHV-6A correlated strongly with dementia scores, neuronal death, and intensity of amyloid plaque.

On the one hand, Aβ neuritic plaques are surrounded by activated microglial cells that could contribute to Aβ phagocytosis and/or compaction [37, 38]. On the other hand, HHV-6A infection might induce Aβ deposition as an innate immune response. Since the major cell component with a role in innate immune response in the brain, we aimed to evaluate the effect of HHV-6A infection on microglia status. In particular, we will evaluate if HHV-6A infection induces Aβ also in microglial cells and the effect on their activation status. Microglial activation and survival are associated with the expression of different molecules as triggering receptor expressed in myeloid cells 2 (TREM2) that seems to perform a pivotal role in the AD-associated immune response [39, 40]. TREM2 is a lipid and lipoprotein sensor that, through its adapter molecule DAP12, supports reactive microgliosis [41]. Furthermore, it has been recently demonstrated that TREM2, interacting with apolipoprotein E (ApoE—a major genetic risk factor for AD), regulates the transcriptional activation of microglial cells [42]. Meanwhile, the expression of tau by microglia cells themselves was also shown to promote their activation [43]. It was found that the expression of tau promoted microglia migration and phagocytosis, and the secretion of several cytokines, including interleukin (IL)-1β, IL-6, IL-10, and tumor necrosis factor-α, suggesting a role of tau in microglial activation. An inappropriate immune response in the brain may be engaged in the neurodestructive processes involved in AD [44]. In fact, chronic microglial activation could improve AD pathology by reducing the Aβ levels in APP-based models. However, the inflammatory response associated with glial activation has been associated with detrimental neurotoxic effects mediated by the release of pro-inflammatory cytokines/chemokines and neurotoxins [45–47]. Increasing pathological Aβ deposits activate glial cells (microglia and astrocytes), lymphocytes, and macrophages, which in turn release large amounts of inflammatory mediators such as cytokines, chemokines, neurotransmitters, and reactive oxygen species (ROS) [48]. Reactive microglia and astrocytes induce neuronal apoptosis and blood-brain barrier (BBB) dysfunction. Next, this process leads to the recruitment of peripheral blood leukocytes (PBL) through the BBB and their active participation in local inflammation in the brain tissue. Leukocytes release more inflammatory factors (cytokines), escalate the inflammatory state, and exacerbate other AD pathologies [49].

We will analyze the effect of HHV-6A infection on microglial cell status, evaluating the expression of genes associated with microglial activation and AD pathogenesis.

The identification of an association between HHV-6A infection and microglial cell status in AD would set the stage for new therapeutic interventions. A positive finding would also lead to a better understanding of the role of microbiota in AD, allowing our study to serve as a “springboard” to a more general use of anti-microbial or immunomodulatory therapies in AD practice.

## Materials and methods

### Study subjects

We recruited five healthy members of societies for retired people for the cognitively normal control group. Only ethnic Italians without known dementia in first degree relatives were included. The study was approved by the Ethics Committee of the University of Ferrara, and the subjects consented to the study in accordance with the Declaration of Helsinki.

### Peripheral blood monocyte-derived microglia

To obtain monocytes (adherent PBMC), the isolated blood cells were cultured in T25 tissue culture flasks (2 × 10^6 to 5 × 10^6 cells/ml) using RPMI-1640 Glutamax medium (Invitrgen, Italy) supplemented with 1% antibiotics/antimycotic (10,000 units/ml penicillin G sodium, 10,000 g/ml streptomycin sulfate, and 25 g/ml Amphotericin B, Invitrogen). After overnight incubation, non-adherent cells will be separated by washing with PBS (Invitrogen) and the fresh adherent cells, which are mainly monocytes (> 90%), will be used for the generation of microglia (M-MG). To induce the differentiation of PBM-microglia, adherent PB will be cultured in 6-well tissue culture plates (Sarstedt; Germany) using RPMI-1640 Glutamax supplemented with1% antibiotic/antimycotic (serum-free condition) and a mixture of human recombinant cytokines, including M-CSF (10 ng/ml; PeproTech, USA), GM-CSF (10 ng/ml; PeproTech), beta-nerve growth factor (NGF-beta 10 ng/ml; PeproTech), and CCL2 (100 ng/ml) for up to 14 day. The generation of PBM-microglia was confirmed by morphology evaluation and immune-phenotype characterization for the expression of substance P with anti-substance P FITC mouse monoclonal antibodies (mAb) and induced the expression of Iba1 by anti-Iba-1 PE mAb [50].

### 3D cultures: microglial spheroid generation

PBM-microglia were seeded in 96-well plate coated with 1.5% agarose to allow the spheroid formation. The microglial spheroids had a 100–250-μm-diameter spheroid at 2–4 days of culture, using 3 × 10^4^ cells/ml medium [51]. Then, spheroids were picked up, transferred into new wells, and used for further experiments.

### HHV-6 infection

The human T cell line J-Jhan was cultured in RPMI-1640 (Gibco BRL, Invitrogen Corporation, Carlsbad, CA, USA) with 10% FCS supplemented with 100 U/ml each of penicillin and streptomycin and maintained at 37 °C in humidified atmosphere of 5% CO_2_. The human immortalized microglial cell line human microglial clone 3 cell line, HMC3, (ATCC®CRL-3304) was maintained in EMEM supplemented with 10% fetal bovine serum supplemented with 100 U/ml each of penicillin and streptomycin and maintained at 37 °C in humidified atmosphere of 5% CO_2_. J-Jhan and HMC3 cells were infected with a HHV-6A (U1102) cell-free inoculum as previously described [52] at a 100:1 m.o.i. The cells were harvested at the experimental time point to perform the analysis.

### HHV-6 analysis

HHV-6 DNA viral load was analyzed by real-time quantitative (qPCR) in duplicate, as described [19].

RNA cell extraction was performed using the RNeasy kit (Qiagen, Hilden, Germany). No DNA contaminated the RNA samples, as shown by control β-actin PCR without retrotranscription [53]. Reverse transcription was performed by the RT2 First strand kit (Qiagen, Hilden, Germany). cDNA aliquots corresponding to 200 ng RNA were used for virus transcription analysis, performed by qPCR detecting the expression of U42 gene, as previously reported [52].

### Immunofluorescence assay

Immunofluorescence for HHV-6 antigen expression was performed with a mAb against glycoprotein gp116 (late antigen) of HHV-6 A and B (ABI, Columbia, MD, USA), as previously described [53]. Microglial cells were stained with anti-substance P FITC (NC1/34HL) mAb anti-Iba-1 PE (1022-5) mAb, anti-TREM2 PE mAb (B-3) (Santa Cruz Biotechnology, USA), anti-Aβ 1-40 (NBP1-44047; Novus Biologicals; Italy), and Aβ 1-42 moAb (ab10148; Abcam; UK).

### APOE and TREM2 expression

Microglial cells, infected with HHV-6A (U1102), were harvested after 1, 3, 7, and 14 days post-infection. Total RNA was extracted (RNeasy kit, Qiagen, Hilden, Germany) and checked for the absence of contaminating DNA and for RNA integrity, and we analyzed only the samples with RIN > 8. RNA reverse transcription was performed as described above. ApoE and TREM2 expression analysis was performed on DNA aliquots corresponding to 200 ng RNA using Applied Biosystems gene expression analysis (Hs00171168_m1 and Hs01003898_m1, respectively) [54].

### Cytokine ELISA assay

IL-1α, IL-1β, IL-6, IL-10, and tumor necrosis factor-α levels in microglial culture supernatants were evaluated by single ELISA kit assays (myBiosource, USA) following the customer’s protocols.

### Tau ELISA assay

Total tau and phosphorylated (p) tau (Threonine 181, T181) were analyzed by ELISA assay (KHB0041 and KHO0631, respectively; Invitrogen; Italy) on cell lysates. Briefly, cell lysates were obtained using a specific lysis buffer prepared adding to RIPA Buffer 1X protease inhibitor cocktail (Roche; Italy), 1% Triton X-100 (Sigma), 1% sodium orthovanadate (Sigma; UK), and 1% PMSF (Sigma). Cell lysates were quantified by the Bradford assay (Biorad; Italy), and then, 20 μg of the total lysate was diluted in 50 or 100 μl of the specific ELISA diluent and seeded into pre-coated wells. The presence of both total tau and ptau (T181) in the samples was revealed by colorimetric reaction and read at 450 nm, and concentration determined through interpolation to standard curve and reported as picograms per milliliter.

### Cell migration assay system

Test J-Jhan cells were plated on the bottom wells (2 × 10^5^ cells/well) of 24 multiwell plates (Falcon; USA) into which FluoroBlock cell culture inserts (BD Biosciences; USA) were to be inserted. These inserts are designed for the plating of cells on a membrane which contains pores of defined size. The base of the membrane blocks all fluorescence transmission, such that when using live cell fluorescence analysis with an inverted microscope, any fluorescence signal originates only from cells that migrated through the pores onto the bottom side of the membrane. We used 8 μm pores. The bottom cell monolayers in the 24-well plates were mock infected or infected with HHV-6A (U1102) cell-free inoculum as previously described [52] at a 100:1 m.o.i. After 1 h of adsorption in serum-free media, the inoculum was removed, and medium containing 2% FBS was added. The inserts, which had been separately plated with HMC3 cells, pre-stained with Syto9 label (Thermo Fisher Scientific; Italy), at a density of 2 × 10^4^ cells/insert, were then placed into the 24-well plates containing the mock infected or infected monolayers. At different times after initial infection, the inserts were removed and analyzed by fluorescence microscopy. Average total Syto 9 staining (pixel density) of target cells after thresholding removed the defined background signal. Values were obtained from three random low-power fields for each condition in each of the duplicated samples.

### Statistical analysis

Data were analyzed by using Student’s *t* test (Stat View software (SAS Institute Inc)). Statistical significance was assumed for *p* < 0.05 (two tailed).

### Data availability

Data are available upon request.

## Results

### Microglial cells are permissive to HHV-6A infection

**Fig. 1:**
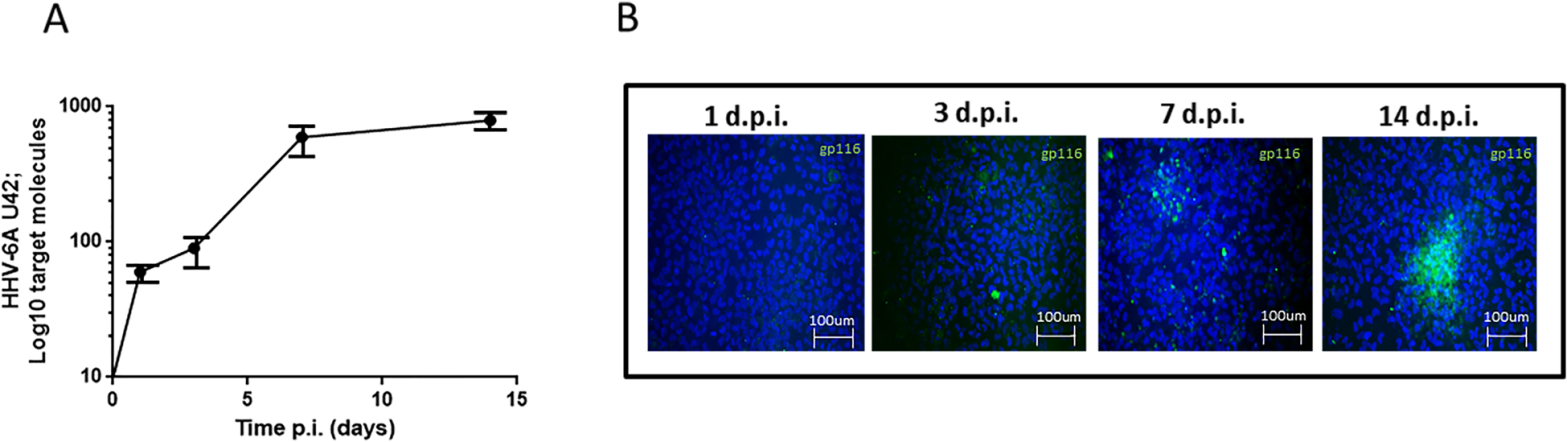
HHV-6A infection of microglial cells. **a** Microglial cells were infected at a multiplicity of infection of 100 genome equivalent/cell. Virus transcription (RNA) was evaluated by RT-qPCR performed on the U42 virus gene, at 1, 3, 7, and 14 d.p.i., as already detailed. Results are expressed in log target molecules referred to duplicates of 2 independent assays. **b** HHV-6A-infected microglial cells (m.o.i. 100:1; 1, 3, 7, 14 d.p.i.) were stained with Hoechst and anti-gp116 FITC moAb. Images were taken in fluorescence (Nikon Eclipse TE2000S) equipped with a digital camera. Original magnification × 20

We evaluated the ability of HHV-6A to infect microglial cells. Microglia were seeded at 60–70% confluence and infected with HHV-6A cell-free inoculum with a m.o.i. of 100:1. HHV-6A infection was evaluated at 1, 3, 7, and 14 days post-infection (d.p.i.) by performing RT-qPCR for immediate early (IE) U42 viral mRNA and immunofluorescent staining for the expression of HHV-6A gp116 late viral antigen. As shown in Fig. 1a, we observed an increase in U42 IE viral gene expression that is maintained during the 14 d.p.i. Similarly, the immunofluorescence of gp116 confirmed microglial permissivity to HHV-6A infection (Fig. 1b). The results obtained by gp116 immunofluorescence showed a temporal shift with respect to RT-qPCR data on the IE viral gene, due to the time lapse between IE and late gene expression.

### HHV-6A infection of microglial cells induces beta-amyloid expression

**Fig. 2:**
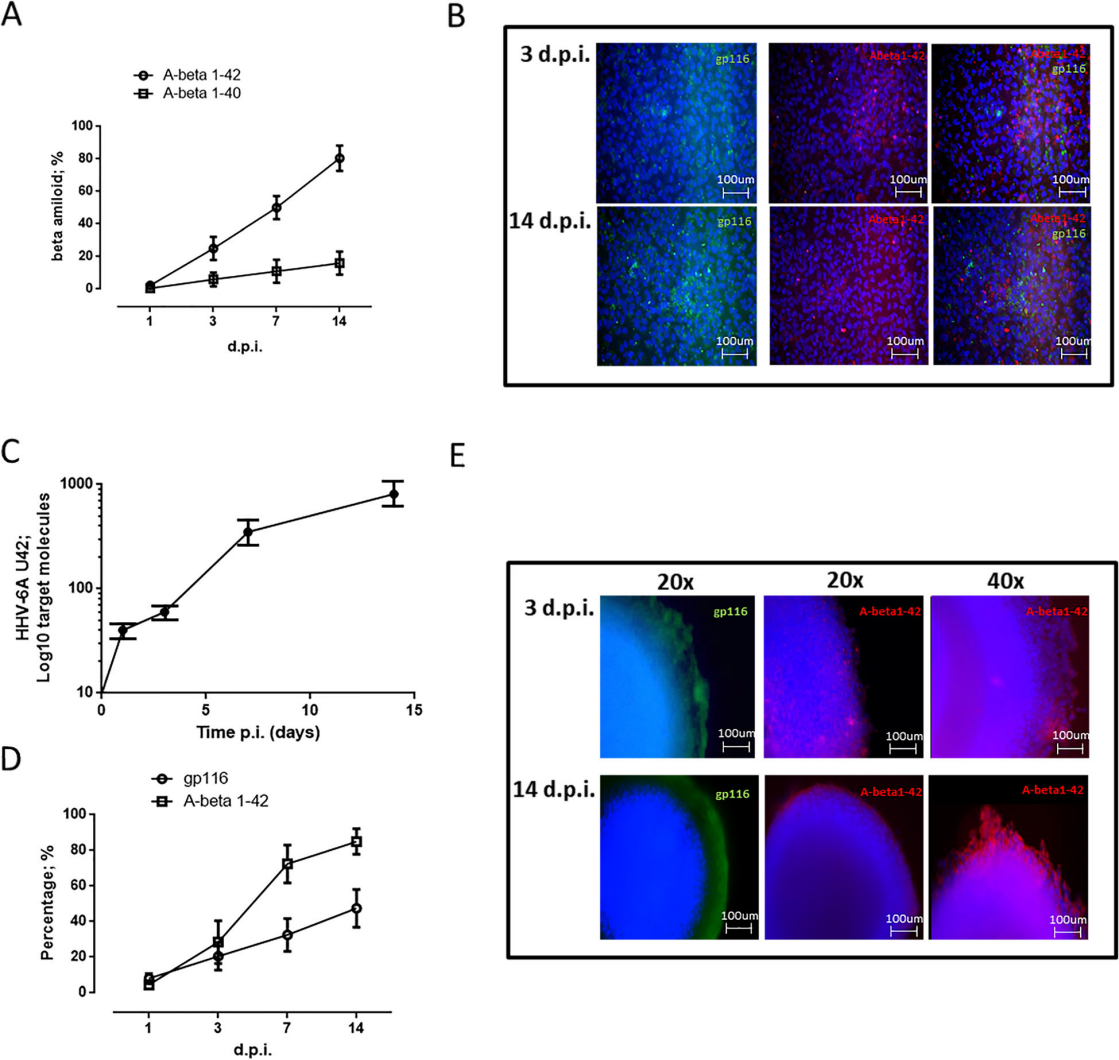
Beta amyloid expression in HHV-6-infected microglial cells. **a** Expression of the two Abeta isoforms more associated to AD (Aβ1-40 and Aβ1-42) was evaluated in monolayer microglial cells infected at a multiplicity of infection of 100 genome equivalent/cell at 1, 3, 7, and 14 d.p.i. The results are reported as percentage of the isoform on the total expression. **b** HHV-6A-infected microglial cells (m.o.i. 100:1; 3, 14 d.p.i.) were stained with Hoechst, anti-gp116 FITC, and anti Abeta 1-42 PE moAbs. Images were taken in fluorescence (Nikon Eclipse TE2000S) equipped with a digital camera. Original magnification × 20. Spheroid 3D models obtained with PBM-microglial cells from healthy subjects. **c** Microglial cells were infected at a multiplicity of infection of 100 genome equivalent/cell. Virus transcription (RNA) was evaluated by RT-qPCR performed on U42 virus gene, at 1, 3, 7, and 14 d.p.i., as already detailed. Results are expressed in log target molecules referred to duplicates of 2 independent assays. **d** Expression of Aβ1-42 and gp116 was evaluated in spheroid 3D microglial cells infected at a multiplicity of infection of 100 genome equivalent/cell at 1, 3, 7, and 14 d.p.i. The results are reported as percentage of the isoform on the total expression. **e** HHV-6A-infected spheroid 3D microglial cells (m.o.i. 100:1; 3; 14 d.p.i.) [14] were stained with Hoechst, anti-gp116 FITC, and anti Abeta 1-42 PE moAbs. Images were taken in fluorescence (Nikon Eclipse TE2000S) equipped with a digital camera. Original magnification: × 20, left panels; × 40, right panels

Aβ is the major constituent of neural plaques, representing one of the more important molecules associated with AD pathogenesis. It has been shown that the presence of HSV-1 and HHV-6 rapidly induces amyloid plaque production within 24 to 48 h [11, 55]. These results support the hypothesis that Aβ deposition and fibrillization might be an innate immune response to pathogens which could protect the brain under normal circumstances. For this reason, we evaluated the effect of HHV-6A on the expression of the two Aβ isoforms more associated with AD (Aβ1-40 and Aβ1-42) [56]. We observed increased expression of Aβ1-42 and a slight increase in expression of Aβ1-40 during HHV-6A infection (Fig. 2a). Interestingly, the immunofluorescence analysis showed a co-localization of gp116 late viral antigen with Aβ1-42 protein expression (Fig. 2b), suggesting a direct effect of HHV-6A infection on Aβ1-42 induction.

As a proof of principle, we infected spheroid 3D models, which were created using PBM-microglial cells from healthy subjects, with HHV-6A. We characterized the PBM-microglia, evaluating the levels of substance P (known to exacerbate neuroinflammation) and Iba-1 (ionized calcium binding adaptor molecule 1; a microglia-specific marker). We observed an increased expression of both substance P and intracellular Iba-1, suggestive of microglial phenotype (Additional file 1: Figure S1A).

HHV-6A spheroid infection was monitored by gp116 expression. We confirmed the permissivity of microglial cells to HHV-6A infection, as documented by the increased transcription of the U42 IE viral gene and the expression of gp116 late viral antigen (Fig. 2c–e). Analysis also showed increased Aβ1-42 expression (Fig. 2d) following the increase in gp116 expression. Interestingly, with a higher magnification, it is possible to observe Aβ deposition, which is evident at 14 d.p.i. (Fig. 2e, left panel).

### HHV-6A infection induces microglia activation status

**Fig. 3:**
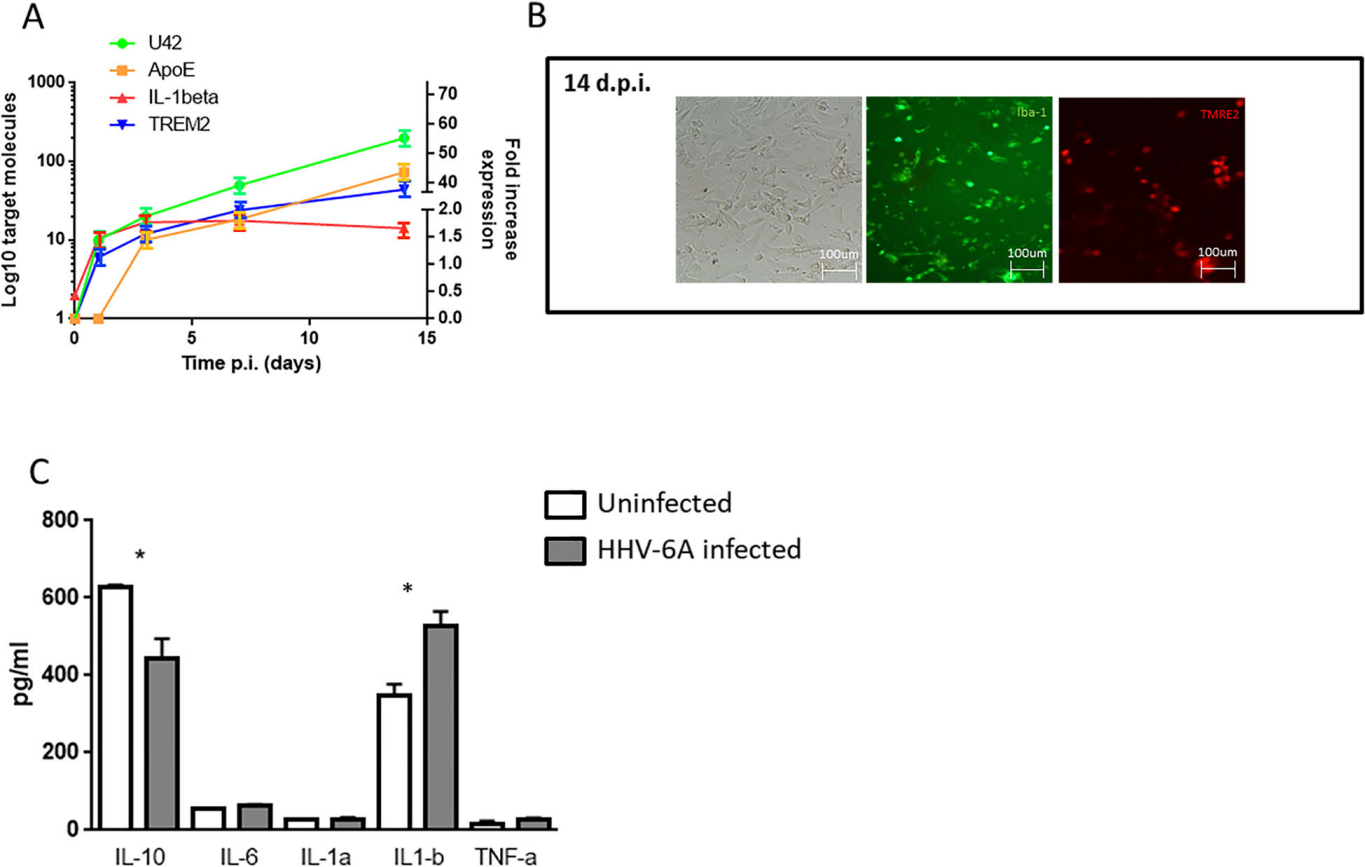
**a** mRNA apoE, IL-1beta, and TREM2 expression was evaluated in microglial cells at 1, 3, 7, and 14 d.p.i. **b** HHV-6A-infected microglial cells (m.o.i. 100:1; 14 d.p.i.) were stained with anti-Iba-1 FITC and TREM2 PE moAbs. Images were taken in bright field (*left panel*) or fluorescence (*right panels*) (Nikon Eclipse TE2000S) equipped with a digital camera. Original magnification × 20. **c** IL-1α, IL-1β, IL-6, IL-10, and tumor necrosis factor-α expression in uninfected (white histogram) and in HHV-6-infected microglial cells (gray histogram). *p value < 0.0001, obtained by Student’s *t* test. Each experiment was performed in triplicate

Microglial activation status might be induced by HHV-6A infection. We evaluated the expression of TREM2, a marker of microglial reactive status, in microglial cells infected with HHV-6A. We observed a significant increase in TREM2 expression, with a 40-fold increase at 14 d.p.i. (Fig. 3a, b). Since it has been recently demonstrated that TREM2, interacting with ApoE, regulates the transcriptional activation of microglial cells [42], we analyzed also ApoE expression in microglial cells. Figure 1c shows that HHV-6A infection also induces ApoE expression in microglial cells. The increase in ApoE expression was 2-fold at 3 d.p.i. and reached a 45-fold increase at 14 d.p.i. The evaluation of pro-inflammatory and anti-inflammatory cytokines (IL-1α, IL-1β, IL-6, IL-10, tumor necrosis factor-α) showed a significant decrease in IL-10 expression (*p* = 0.012; Student’s *t* test) and an increase in IL-1beta expression (*p* < 0.001; Student’s *t* test) (Fig. 3c). Since IL-1beta is detectable at abnormal levels in AD, with a dose-dependent correlation between ApoE and the levels of pro-inflammatory cytokines [57], we correlated IL-1beta and ApoE expression with HHV-6A infection. The analysis of IL-1beta expression showed a significant increase during HHV-6A infection, with a 2-fold increase at 3 d.p.i., after which it plateaued (Fig. 3a). During the first 6 d.p.i., the IL-1beta expression followed ApoE increase (Fig. 3a).

### HHV-6A infection of microglial cells induces tau phosphorylation

**Fig. 4:**
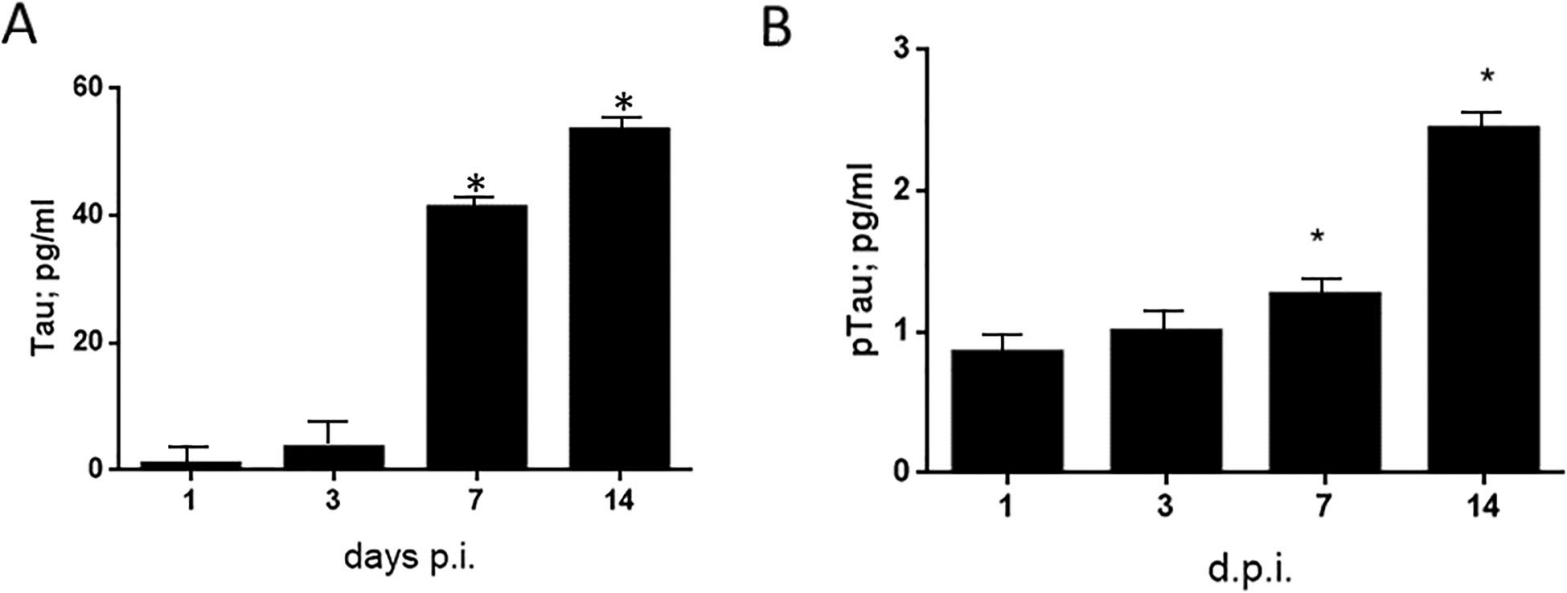
Tau and phosphorylated tau (ptau) expression in HHV-6-infected microglial cells. **a** Expression of tau and **b** phosphorylated tau (ptau) was evaluated in monolayer microglial cells infected at a multiplicity of infection of 100 genome equivalent/cell at 1, 3, 7, and 14 d.p.i. The results are reported as mean ± SD pg/ml. *p value < 0.01, obtained by Student’s t test. Each experiment was performed in triplicate

Tau is one of the microtubule-associated proteins that regulate the stability of tubulin assemblies. In AD brains, tau is accumulated in a hyper-phosphorylated state in the pathological inclusions [58, 59]. The expression of tau by microglial cells themselves was also shown to promote their activation and secretion of several cytokines [43]. We investigated total-tau and p-tau (T181) levels in healthy donor PBM-microglial cells infected with HHV-6A. HHV-6A infection was associated with an increase of both total-tau (Fig. 4a, *p* < 0.0001, Student’s *t* test) and p-tau (T181) (Fig. 4b, *p* < 0.0001, Student’s *t* test), particularly 7 and 14 d.p.i.

### HHV-6A infection induces microglial cell migration

**Fig. 5:**
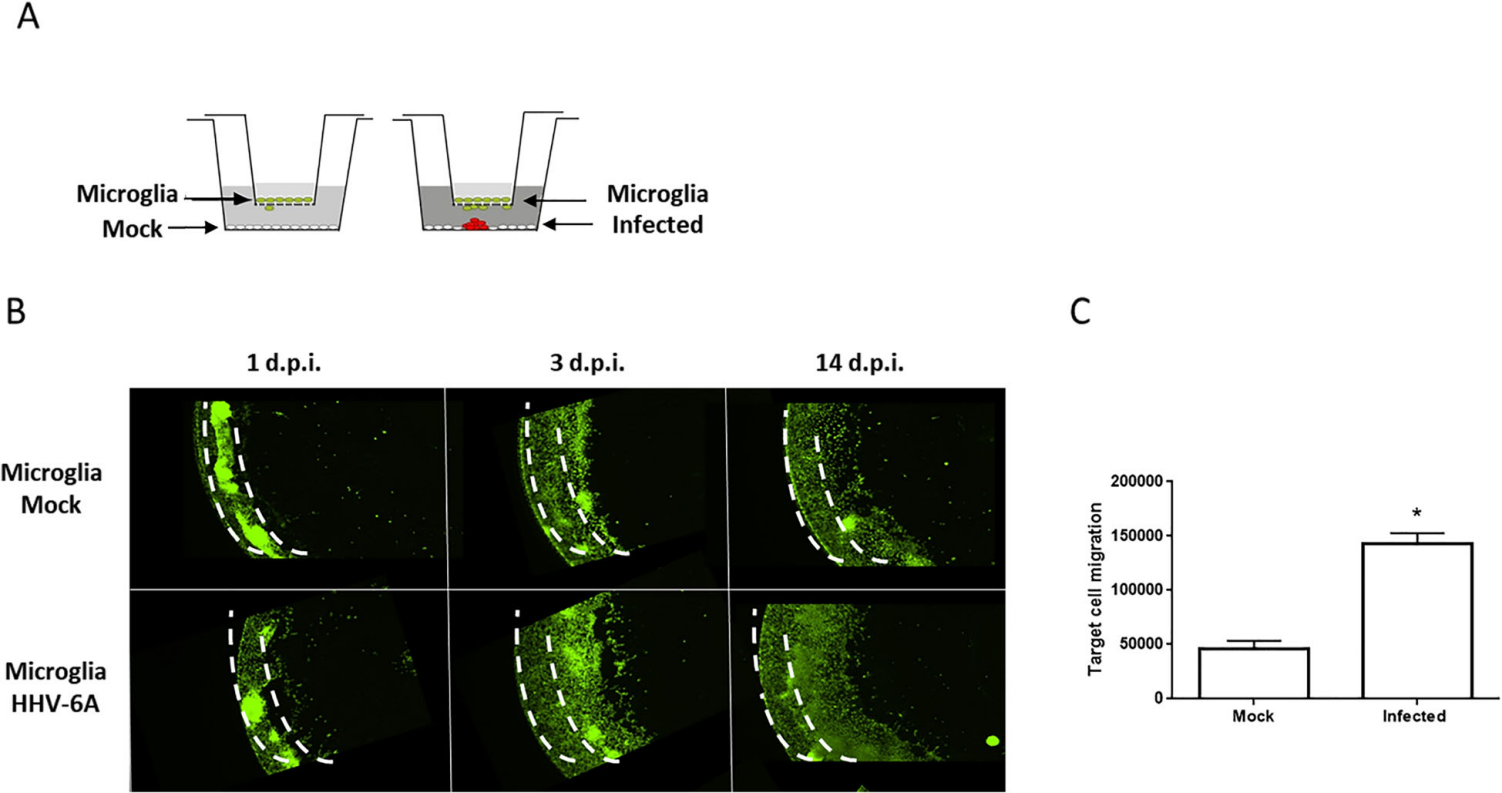
Cell migration of HHV-6-infected microglial cells. **a** Cell migration assay system where target microglial cells were plated in the upper chamber insert on a membrane support with defined 8-μm pores. The insert was then placed in a dish of test cells (lower chamber) that were either mock infected or infected with 100:1 m.o.i. **b** Representative images of target microglial cells stained with Syto 9. **c** Average total Syto 9 staining (pixel density) of target cells after thresholding to remove the defined background signal (image in Fig. 4b). Values were obtained from three random low-power fields for each condition in each of the duplicated samples. The experiment was repeated three times with similar results

Using a cell migration assay system (see the “Materials and methods” section), we assessed whether there was evidence that HHV-6A infection could induce microglial cell migration at the site of infection. Target microglial cells were plated in the upper chamber insert on a membrane support with defined 8-μm pores (Fig. 5a). The insert was then placed in a dish of test cells (lower chamber) that were either mock infected or infected with 100:1 m.o.i. We confirmed that the upper target cells remained uninfected (Additional file 1: Figure S1B). Cell migration was then monitored by the presence of cells on the lower side of the membrane support by live cell staining. The results demonstrated a very clear increase in target cell migration with infected test cells compared to uninfected test cells (Fig. 5b, c). This could be readily seen in the images of target cell migration (Fig. 5b) and in the quantitation of the data (Fig. 5c). Identical results were obtained in repeated experiments and in experiments using inserts with a 3-μm pore size instead of 8 μm (data not shown). This system suggests a paracrine effect of HHV-6A infection, since the cells are not in direct contact with one another.

## Discussion

There is increasing evidence of a possible role for viral infections in the progression of AD, in addition to known risk factors. To date, the most studied viruses in correlation to AD development are herpesviruses [60], since they have the ability to undergo latency and then reactivate, causing persistent infection and neuroinflammation, which is observed in AD [61]. Recent findings have shown that infections may induce Aβ production and deposition in the brain with an anti-microbial activity [11]. Aβ fibrilization pathway seems to be an innate immune response that protects against viral, fungal, and bacterial infections. Aβ oligomers bind herpesvirus surface glycoproteins, accelerating β-amyloid deposition and leading to protective viral entrapment activity [11, 12]. One such infection is that of HHV-6, which has been examined for its potential role in AD pathogenesis [10, 13].

The aim of this study was to evaluate the role of HHV-6A infection of CNS accessory cells (microglia) in AD pathogenesis. We developed an in vitro model of the human microglial cells, using both monolayer and spheroid cultures, in order to evaluate the effects of HHV-6A infection on Aβ expression and on activation status.

HHV-6A can productively infect microglial cells both in monolayer and spheroid conformation, as confirmed by the increased transcription and expression of viral proteins (Figs. 1a; 2c, e; and 3a).

HHV-6A predominantly induces expression of Aβ1-42 (Fig. 2a, b), which is co-localized with HHV-6A infection sites (Fig. 2b). The recent discovery that Aβ is an anti-microbial peptide (AMP) acting against bacteria, fungi, and viruses gives increased credence to an infection hypothesis in the etiology of AD [62–64]. The production of Aβ as an AMP will be beneficial on first microbial challenge but will become progressively detrimental as the infection becomes chronic and reactivates from time to time [65]. It has been shown that HSV-1 and HHV-6 catalyze the aggregation of the amyloid β-peptide (Aβ42), a major constituent of amyloid plaques in Alzheimer’s disease, in vitro and in animal models. The viral protein corona seems to be critical for viral-host interactions and illustrates a mechanistic convergence between viral and amyloid pathologies [55].

HHV-6A infection induced the activation of microglial cells as shown by the increased expression of TREM2. It is already known that a soluble form of TREM2 (sTREM2) derived from proteolytic cleavage of the cell surface receptor is increased in the preclinical stages of AD, positively correlates with the amounts of total and phosphorylated tau in the cerebrospinal fluid, promotes microglial survival in a PI3K/Akt-dependent manner, and stimulates the production of inflammatory cytokines depending on NF-κB. The TREM2-ApoE pathway is important for facilitating the microglial response to damage in the brain, and a functional consequence of activation of the TREM2-ApoE pathway is that microglia lose the ability to regulate brain homeostasis [42]. Previous studies suggested a link between virus-perturbed lipids and TREM2 that modulates pathogenesis upon viral infection [66], suggesting a possible link between the ApoE-TREM2 pathway and activation of HHV-6A-infected microglial cells. We observed a significant increase in ApoE expression 3 d.p.i. (Fig. 3a), confirming our previous data on ApoE [54]. Similarly, we observed an increase in IL-1beta expression, suggesting the involvement of HHV-6A in microglial activation and IL-1beta secretion, which may be involved in the induction of CNS neuroinflammation in AD [60]. Many studies now point to the involvement of neuroinflammation playing a fundamental role in the progression of the neuropathological changes that are observed in AD [67], where IL-1 elevated levels seem to be responsible for the increased APP production and Aβ load [68]. Meanwhile, Parajuli and coauthors described that soluble oligomeric amyloid-β (oAβ) increased the processing of pro-IL-1β into mature IL-1β in microglia via ROS-dependent activation of NLRP3 inflammation [69]. In addition, Aβ has been reported to activate microglia, leading to increased synthesis and release of neurotoxic secretory products, pro-inflammatory cytokines such as IL-1β, and ROS [70]. Based on these findings, we can hypothesize a feedback loop between Aβ deposits and IL-1β expression in AD patients.

The analysis of total-tau and p-tau (T181) showed an increase in both total and phosphorylated proteins in the presence of HHV-6A infection. It has been previously shown that viral infection-induced acute or chronic inflammation significantly exacerbates tau pathological characteristics, and that the chronic inflammation leads to impaired spatial memory in mice [71].

HHV-6A infection can induce microglial cell migration to the site of infection. We believe these results indicate that HHV-6A-infected cells release a soluble mediator that can stimulate microglial cell migration. This paracrine effect of HHV-6A infection might in part explain the activation status of microglial cells in AD patients. Microglia, the resident innate immune cells in the brain, have long been implicated in the pathology of neurodegenerative diseases [72]. Accumulating evidence points to activated microglia as a chronic source of multiple neurotoxic factors, including IL-1beta, driving progressive neuron damage. Microglia can become chronically activated by stimuli (e.g., HHV-6A infection), resulting in chronic neuroinflammation exacerbated by the TREM2-ApoE pathway and IL-1beta expression. Interestingly, Liddelow and coauthors showed that the activation of microglia might precede the activation of astrocytes, which in turn appear to be responsible for neuronal cell death [73].

## Conclusions

In conclusion, this study showed the permissivity of microglial cells to HHV-6A infection, supporting the hypothesis of HHV-6A involvement in AD pathogenesis. Its ability to induce Aβ1-42 supports the role of this molecule as anti-microbial agent. TREM2, ApoE, IL-1beta, and p-tau (T181) are suggestive of a direct effect of HHV-6A infection on microglial cell activation that should be evaluated carefully. Moreover, the ability of HHV-6A infection to induce migration of microglial cells strengthens the hypothesis of HHV-6A playing an important role in AD. Further studies on the mechanism at the basis of HHV-6A-mediated control of the microglial gene expression profile are needed to identify potential antiviral therapies for AD management.

## Supplementary information

**Additional file 1: Figure S1A.** Characterization of PBM-microglial cells. At Day 12, a ramified morphology predominated, and cells increased the expression of Substance P (anti Substance P FITC) and induced the expression of Iba1 (anti-Iba-1 PE). Three independent donors with triplicate staining were analyzed with similar results. **B**. Virus transcription (RNA) was evaluated by RT-qPCR performed on U42 virus gene, at 1, 3, 7, 14 d.p.i. in target microglial cells (Target) and in HHV-6A-infected (HHV-6A) JJhan cells.

## Data Availability

Data are available upon request.
