# Supplementary figures and images for: HHV-6A infection induces amyloid-beta expression and activation of microglial cells
Source: Alzheimers Res Ther. 2019 Dec 12;11:104. doi: 10.1186/s13195-019-0552-6 (PMC6909659; doi:10.1186/s13195-019-0552-6)

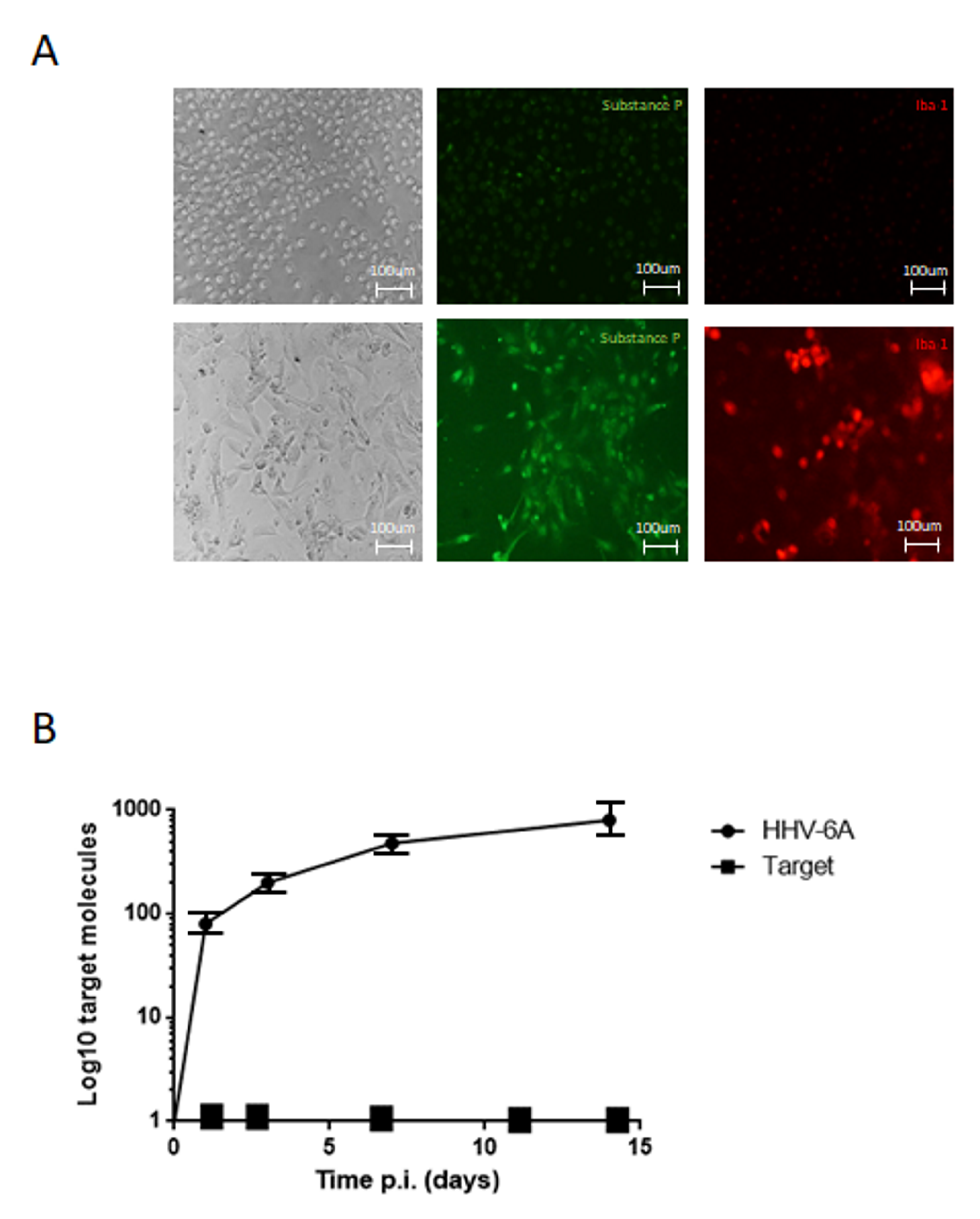

Supplement: Supplementary file 1 — Additional file 1: Figure S1A. Characterization of PBM-microglial cells. At Day 12, a ramified morphology predominated, and cells increased the expression of Substance P (anti Substance P FITC) and induced the expression of Iba1 (anti-Iba-1 PE). Three independent donors with triplicate staining were analyzed with similar results. B. Virus transcription (RNA) was evaluated by RT-qPCR performed on U42 virus gene, at 1, 3, 7, 14 d.p.i. in target microglial cells (Target) and in HHV-6A-infected (HHV-6A) JJhan cells. [file 13195_2019_552_MOESM1_ESM.tif]
